# Obesity and Age-Related Changes in the Brain of the Zucker *Lepr* *^fa/fa^* Rats

**DOI:** 10.3390/nu12051356

**Published:** 2020-05-09

**Authors:** Daniele Tomassoni, Ilenia Martinelli, Michele Moruzzi, Maria Vittoria Micioni Di Bonaventura, Carlo Cifani, Francesco Amenta, Seyed Khosrow Tayebati

**Affiliations:** 1 School of Biosciences and Veterinary Medicine, University of Camerino, Via Gentile III da Varano, 62032 Camerino, Italy; daniele.tomassoni@unicam.it; 2 School of Pharmacy, University of Camerino, Via Madonna delle Carceri, 9, 62032 Camerino, Italy; ilenia.martinelli@unicam.it (I.M.); mariavittoria.micioni@unicam.it (M.V.M.D.B.); francesco.amenta@unicam.it (F.A.); 3 Department of Medicine, University of Leipzig, Liebigstraße 21, 04103 Leipzig, Germany; michele.moruzzi@medizin.uni-leipzig.de

**Keywords:** brain alterations, diabetes, metabolic syndrome, neuromorphology, obesity, Zucker rats

## Abstract

Metabolic syndrome (MetS) is an association between obesity, dyslipidemia, hyperglycemia, hypertension, and insulin resistance. A relationship between MetS and vascular dementia was hypothesized. The purpose of this work is to investigate brain microanatomy alterations in obese Zucker rats (OZRs), as a model of MetS, compared to their counterparts lean Zucker rats (LZRs). 12-, 16-, and 20-weeks-old male OZRs and LZRs were studied. General physiological parameters and blood values were measured. Immunochemical and immunohistochemical techniques were applied to analyze the brain alterations. The morphology of nerve cells and axons, astrocytes and microglia were investigated. The blood–brain barrier (BBB) changes occurring in OZRs were assessed as well using aquaporin-4 (AQP4) and glucose transporter protein-1 (GLUT1) as markers. Body weight gain, hypertension, hyperglycemia, and hyperlipidemia were found in OZRs compared to LZRs. In the frontal cortex and hippocampus, a decrease of neurons was noticeable in the older obese rats in comparison to their age-matched lean counterparts. In OZRs, a reduction of neurofilament immunoreaction and gliosis was observed. The BBB of older OZRs revealed an increased expression of AQP4 likely related to the development of edema. A down-regulation of GLUT1 was found in OZRs of 12 weeks of age, whereas it increased in older OZRs. The behavioral analysis revealed cognitive alterations in 20-week-old OZRs. Based on these results, the OZRs may be useful for understanding the mechanisms through which obesity and related metabolic alterations induce neurodegeneration.

## 1. Introduction

High food intake, low physical exercise, and impaired metabolic activities are responsible for energy imbalance that leads to obesity. Metabolic syndrome (MetS) is a multifactorial syndrome caused by an insulin resistance followed by abnormal adipose tissue deposition and function. Moreover, arterial hypertension, hyperglycemia, hypertriglyceridemia, HDL cholesterol reduction, and abdominal obesity are common clinical manifestations. MetS is a hazard factor for developing diabetes, fatty liver, coronary heart disease, oncological pathologies, and brain injury [1,2].

Epidemiological studies consider MetS and its distinctive aspects as a cause of the increase of cardiovascular and cerebrovascular diseases, as well as mortality [3,4,5]. According to the World Health Organization (WHO), obesity is a pathology with a total body fat percentage greater than 35% in women and 25% in men [6]. Excessive body mass index is related to the appearance of cardiovascular risk factors, for example, dyslipidemia, hypertension, insulin resistance, and diabetes. These factors lead to cerebrovascular, renal, and hepatic diseases [7,8,9,10].

Other studies report that overweight and/or obesity could elicit brain damage, leading to dementia or neurodegeneration [11,12]. A link between obesity-related complications and Alzheimer’s disease (AD) was reported [13,14,15]. Neuropathologies triggered by MetS often result in loss of integrity of the blood–brain barrier (BBB). This induces alterations of glial and neuronal cells, causing hormonal imbalance, increased immune sensitivity, or cognitive impairment. Inflammatory effects of MetS have been linked to neurodegenerative diseases [16]. Neuroinflammation, increased formation of free radicals, altered neurotrophic factors, and reduced insulin transport into the brain were found in subjects affected by MetS [17].

The onset and development of obesity is attributed to both genetic and environmental factors [18], but binge eating and sedentary lifestyle are implicated in the advancement toward depression and synaptic impairments [19,20]. This indicates that excessive food intake can negatively influence cerebral activities. The mechanisms responsible for the increased susceptibility of nervous system changes in obesity were not clarified yet.

As the MetS is a multifactorial disease, it is not easy to find adequate experimental models to understand this pathology. The obese Zucker rats (OZRs) are homozygous for the *fa* allele of the leptin receptors gene. These rats are mainly used as obesity experimental model, but they also present alterations similar to human MetS, which makes them the most suitable rats strain to study the MetS [21,22]. OZRs become noticeably obese since 3–5 weeks of age, and since 14 weeks of age, their body composition is over 40% lipid compared to the littermate lean Zucker rats (LZRs).

The present study is designed to identify the relationships between MetS and the possible nervous system alterations in the brain of OZRs. The analysis was done by investigating possible neuronal alterations, glial activation in neuroinflammatory processes, and the integrity of blood–brain barrier (BBB) of OZRs and littermate controls LZRs at 12-, 16-, and 20-weeks of age in frontal cortex and hippocampus as the main brain areas involved in cognition.

## 2. Materials and Methods

### 2.1. Animals, Behavioral Tests, and Tissue Treatment

Male OZRs (*n* = 18) and their littermate LZRs (*n* = 18) were purchased from Harlan (San Pietro al Natisone, UD, Italy), housed (1 per cage) starting from 8 weeks of age under 12:12 h light/dark cycle with *ad libitum* access to food and water. They were fed with the same diet (Mucedola 4RF18 MICE and RATS Long Term Maintenance, containing 16% protein, 2.5% fat, and 7.5% max fiber and other nutritional additives). They stayed in a room with a temperature of around 20–22 °C and 45–55% humidity. After two weeks of housing, the OZRs and LZRs were randomized in three different groups, depending on the different age of sacrifice.

Taking into account that rodents are social species and the isolation might influence the experimental results [23,24], in this study the rats were not totally isolated, they could see, smell, hear, and maintain contact with each other, as van Loo et al. [25] and Krohn et al. [26] suggested. The rats were immediately housed in individual cages to avoid the possibility of food consumption reduction in group-housed rats [27].

Finally, they were sacrificed at 12 weeks (*n* = 6, for each group), 16 weeks (*n* = 6, for each group), and 20 weeks of age (*n* = 6, for each group). The number of rats for each experimental group was identified based on a previous study [28,29], considering an increase of the astrocytes area (effects size ~40%, a standard deviation ~15%, level of significance 5%, and power of test 0.95). Origin 9.1 software (OriginLab, Northampton, MA, USA) was used for statistical analysis.

All procedures regarding rats were in agreement with the Institutional Guidelines and complied with the Italian Ministry of Health (D.Lgs. 116/92–Art. 7 Prot. N. 6198/2011) and associated guidelines from European Communities Council Directive (n. 86/609/CEE) governing animal welfare and protection. The identification code is: 6198. Bodyweight and food intake were measured every day while the blood pressure was measured once a week, by a tail-cuff apparatus and sphygmomanometer (Model: GIMA Italy, B3Plus). Blood samples were collected in the morning (9:00 AM) during the light phase from the caudal artery every two weeks in fasted rats for the determination of the levels of glucose, insulin, triglycerides, and total cholesterol. Blood samples (1 ml) were collected in a heparin tube and centrifuged at 3000 rpm for 10 min. Samples were stored at 4 °C and delivered within 24 h to the “Fioroni, Diagnostica Veterinaria” laboratory (San Benedetto del Tronto, AP, Italy) for the biochemical assay of blood parameters. The glycemia determination was performed by hexokinase method, the total cholesterol analysis was performed by the Chod-pap method, and the triglycerides were determined by the Gpo-pap method. For the insulin determination “Rat Insulin Enzyme Immunoassay kit” (Bertin Pharma, CAT. #A05105-Spi-Bio) was used.

The open field test measures rats’ locomotor activity and anxiety-like behavior in different animal group. The text was performed one week before the sacrifice. The open-field box consisted of a square box made of plastic with the following dimensions: 43 × 43 floor area and 25 × 25 cm virtual central zone (Med Associates, St Albans, Vermont, USA) [30]. The overall activity in the box was measured for 10 min to quantify the spontaneous locomotor parameters of the animal placed in the cage. The movements were registered automatically by interruption of two orthogonal light beams that were connected to the software. Locomotion counts were registered when the low row of photocells was interrupted. Increased locomotor activity in the entire field was considered a sign of behavioral arousal, whereas reduced locomotor activity in the central zone and numbers of entries into the central zone were considered signs of increased emotionality, anxiety, or fear in mice and rats [31,32,33]

The passive avoidance test measures rats’ capacity to learn and to keep away from unpleasant electric shock. This test was performed using an apparatus composed of two chambers with the same dimensions (20 cm × 20 cm × 40 cm), connected with a rectangular door. The rat is placed into the chamber with the light, and the time spent to move into the dark chamber is recorded. During the training, after entry into the dark side, the rat receives a mild foot shock (1.24 mA 3 s) and it is removed from the box. Electric shock was delivered to the grid floor by a constant current generator (Med Associates, St Albans, Vermont, USA) [30]. For the retention assessment, after 72 h the rat is then placed back into the same context (cage where it received foot shocks) and cross-over latency is measured, up to a maximum of 300 s as the cut-off.

For the sacrifice, rats were anesthetized with an intraperitoneal injection of pentobarbital sodium (50 mg/kg) and sacrificed by decapitation. The brain was cut apart and separated into two halves at the level of medial longitudinal fissure. The frontal cortex and hippocampus areas of the right hemisphere were immediately frozen in dry ice and stored at −80 °C to perform immunochemical techniques. The left hemisphere was fixed in 4% buffered paraformaldehyde solution and processed for paraffin embedding.

Different oxidative stress indicators were evaluated: thiobarbituric acid reactive substances (TBARS) Cayman, Chemical Company, Ann Arbor, MI, USA Cat. No. 10009055; glutathione peroxidase (GPX) activity by Cayman, Chemical Company, Ann Arbor, MI, USA Cat. No. 703102; superoxide dismutase (SOD) activity by Cayman, Chemical Company, Ann Arbor, MI, USA Cat. No.706,002 and the protein oxidation status by the OxyBlot Protein Oxidation Detection Kit (Millipore, USA, Cat. No. S7150).

### 2.2. Western Blot Analysis

Samples of the frontal cortex and hippocampus, taken from six rats for each group, were homogenized in lysis buffer, as previously described [34]. Equal amounts of protein (40 μg) were separated by 6% to 12% SDS polyacrylamide gel, transferred onto nitrocellulose and blotted with the specific primary antibodies at different dilutions, as detailed in Table 1. This step is followed by incubation with the specific HRP-secondary antibodies (Table 1) and the signal from the protein of interest is visualized by incubating the membrane with a chemiluminescent detection reagent. To normalize protein loadings, membranes were stripped and incubated with anti-glyceraldehyde 3-phosphate dehydrogenase (GAPDH) antibody (Table 1). Bands intensities were estimated densitometrically with IAS 2000 image analyzer (Biosystem, Rome, Italy).

### 2.3. Immunohistochemistry

A microtome was used to make thin slices (10 μm) of the left hemisphere of the brain from each rat. Five groups of eight consecutive sagittal sections were attached to poly-l-lysine-coated slides. To highlight the possible morphological alterations, the first of each group of eight consecutive sections cut from the left hemisphere, were stained with a 0.5% cresyl violet. The other six were processed independently for immunohistochemistry using different antibodies at the various dilutions in PBS + TritonX-100 0.3% (PBS-T), as detailed in Table 1. The last for each group was used as a negative control to test the specificity of different antibodies. After incubation with specific biotinylated secondary antibody (Table 1), the product of immunoreaction was revealed by the indirect method through the complex of avidin-biotin-peroxidase. The peroxidase is then developed by the 3,3′-diaminobenzidine (DAB). Sections were viewed under a light microscope (Leica DMR). The images were transferred by a digital camera to an IAS 2000 image analyzer (Biosystem, Rome, Italy) and used for assessing the intensity of the immune reaction. For confocal laser microscopy, the sections were incubated with specific fluorochrome-conjugated secondary antibodies (Table 1), counterstained with 4′,6-diamidino-2-phenylindole, dihydrochloride (DAPI) and viewed using a Nikon mod.C2 plus Confocal Laser Microscope (Nikon, Corporation, Japan).

### 2.4. Morphological Analysis

Sections were analyzed in the frontal cortex and the hippocampal areas CA1 or CA3 and dentate gyrus (stereotaxis coordinates: Lateral 1.40 mm). The density of immunoreactions area occupied by neurofilament 200-kDa (NF) immunohistochemistry was measured by the image analysis protocol previously described [35]. The intensity of aquaporin-4 (AQP4) and glucose transporter 1 (GLUT1) immunostaining was assessed microdensitometrically. In slides processed for anti-glial fibrillary acid protein (GFAP) immunohistochemistry, astrocytes of different layers of frontal cortex and subfields of the hippocampus were selected and their areas were measured by the specific function of the software. Morphological analysis of microglia was carried out in optical microscopy samples. Microglia analysis was performed by capturing images at a 63X magnification, measuring the soma of the cells. Measurements were made blindly by two researchers independently.

### 2.5. Statistical Analysis

Means of different parameters were calculated from single animal data, and group means ± SEM were then derived from single animal values. The significance of differences between means was analyzed by two-way analysis of variance (ANOVA) followed by the Bonferroni multiple range tests as post-hoc test setting *p* < 0.05 value as a significant difference. No outliers were present

## 3. Results 

### 3.1. General and Blood Analysis Indicate a Condition of Dismetabolis Similar to MetS

**Figure 1:**
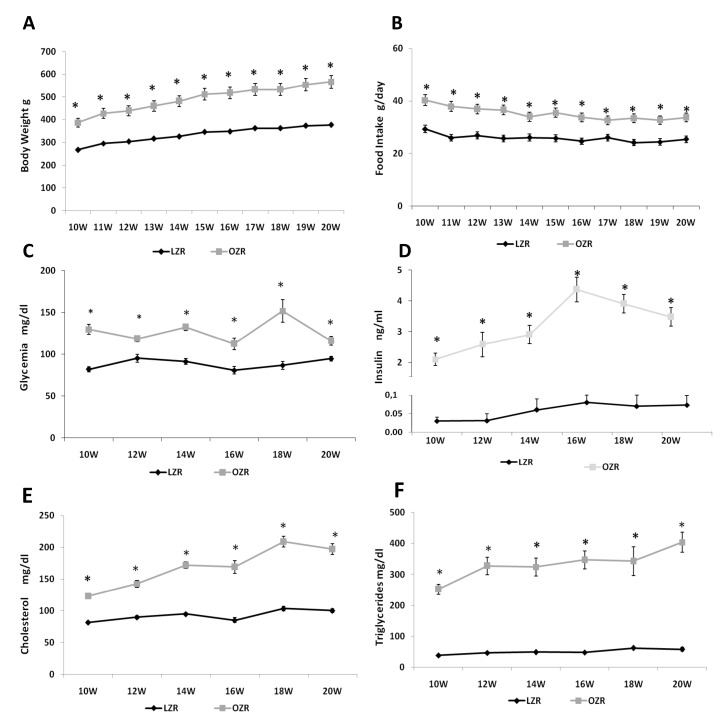
General and blood parameters. Body weight (**A**), food intake (**B**) starting from 10th week of age (first week of housing) to 20th week of age (last sacrifice) for lean Zucker rats (LZR) and obese Zucker rats (OZR). Plasma levels of glucose (**C**), insulin (**D**), total cholesterol (**E**), and triglycerides (**F**) for LZRs and OZRs at different ages. Data are the mean ± SEM. * *p* < 0.05 vs. age-matched LZR.

In the time of study, overall ANOVA showed significant differences between the two strains (*p* < 0.001) for the different weeks (*p* < 0.001). The value of body weight was significantly higher in OZRs than LZRs, starting from 10 weeks of age until 20 weeks of age (Figure 1A). At the same time, overall ANOVA showed that food intake was also higher in OZRs compared to LZRs (*p* < 0.001). The results of post-hoc analysis are shown in Figure 1B, indicating a condition of obesity due to hyperphagia. Before the sacrifice, at 12, 16, and 20 weeks of age, the values of systolic blood pressure were measured again. LZRs systolic blood pressure averaged 103.6 ± 9.1 mmHg at 12 weeks, 104.3 ± 6.4 mmHg at 16 weeks, and 99.8 ± 2.2 mmHg at 20 weeks. In OZRs the systolic blood pressure values were 120.1 ± 13.7 mmHg at 12 weeks, 140.8 ± 5.6 mmHg at 16 weeks (*p* = 0.002 vs. age-matched LZRs), 137.3 ± 4.2 mmHg at 20 weeks of age (*p* = 0.007 vs. age-matched LZRs). Overall ANOVA for the blood parameters analyzed indicate a significant difference (*p* < 0.001) between the two strains. The results of post-hoc analysis of glycemia, insulin, triglycerides, and total cholesterol are summarized in Figure 1. As shown, these parameters were higher in OZRs compared to the LZRs at different ages (Figure 1C–F), indicating a condition of dysmetabolism.

### 3.2. Oxidative Stress Conditions in Brain Areas

**Figure 2:**
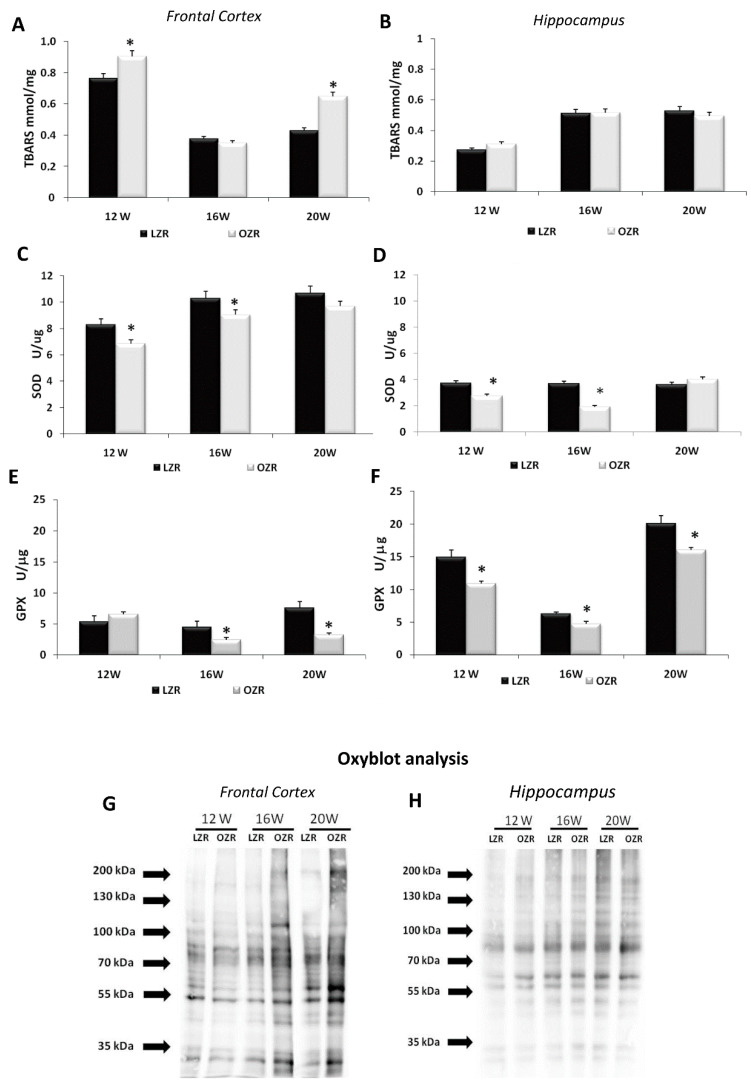
Oxidative stress parameters in the frontal cortex (**A**,**C**,**E**,**G**) and hippocampus (**B**,**D**,**F**,**H**) for LZRs and OZRs of 12-, 16-, and 20-weeks age. The data of thiobarbituric acid reactive substances TBARS level (expressed in mmol/mg of tissue) (**A**,**B**), superoxide dismutase activity SOD (expressed as U/μg of proteins where one unit is the amount of enzyme needed to exhibit 50% dismutation of the superoxide radical) (**C**,**D**), and glutathione peroxidase activity GPX (expressed U/μg where one unit define as the amount of enzyme that will cause the oxidation of 1.0 nmol of NADPH to NADP+ per minute at 25 °C) (**E**,**F**). Data are the mean + SEM. * *p* < 0.05 vs. age-matched LZRs. The two inferior panels represent the results of Oxyblot of proteins extracted from the frontal cortex (**G**) and hippocampus (**H**).

Different oxidative stress markers were analyzed in several brain areas. Lipid peroxidation was evaluated by the TBARS kit that represents a well-established method for screening the levels of malondialdehyde (MDA) as a naturally occurring product of lipid peroxidation [36,37]. Overall ANOVA showed a difference in TBARS concentration between the two strains in the frontal cortex (*p* = 0.008) but not in the hippocampus (*p* = 0.985) as shown in Figure 2. Post-hoc analysis revealed that TBARS concentration in the frontal cortex was higher in 12 and 20 weeks-old OZRs compared to age-matched LZRs (Figure 2A), while in the hippocampus no differences were present between obese and lean rats in different ages (Figure 2B). In the analysis of the antioxidant enzyme SOD, the overall ANOVA showed a significant difference between the two strains both in the frontal cortex (*p* = 0.003) and in the hippocampus (*p* = 0.004). SOD specific activity was decreased in frontal cortex and hippocampus of 12 weeks and 16 weeks-old OZR compared to the age-matched LZRs (Figure 2C,D). A significant decrease in GPX specific activity was found in OZRs compared to the LZR both in the frontal cortex (*p* = 0.004) and in the hippocampus (*p* = 0.001) (Figure 2E,F). Moreover, an increased density of the oxidized proteins was detected in the membrane of the Oxyblot in the OZRs 16- and 20-weeks-old compared to age-matched LZRs (Figure 2G,H), especially in the frontal cortex (Figure 2G).

### 3.3. Neuronal and Glial Markers Analysis Highlighted Neurodegeneration and Gliosis in Obese Rats

**Figure 3:**
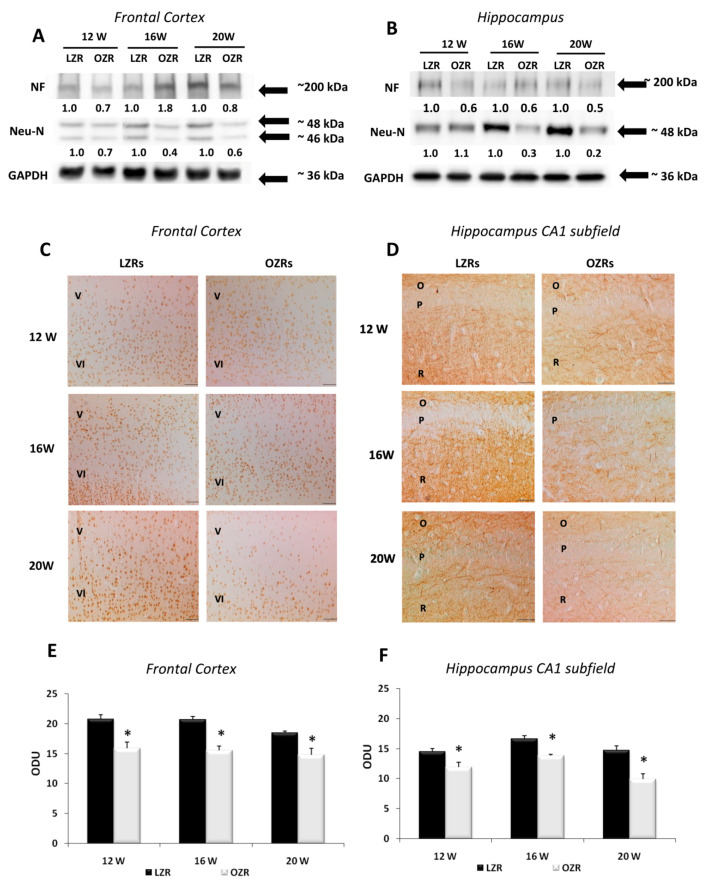
Neuronal markers. Immunochemical analysis of the frontal cortex (**A**) and hippocampus (**B**) processed with different antibodies anti-neurofilament (NF) and anti-neuronal nuclei protein (NeuN). To investigate more than NF and NeuN proteins on the same blot, both membranes of the frontal cortex and hippocampus were stripped and incubated with anti-GAPDH antibody (loading control). The densitometric analysis of bands is expressed as the ratio between the optical density of protein and reference protein (GADPH) where the value for LZRs of different ages is set as 1. Blots are representative of one of three separate experiments. (**C**) Sections of the frontal cortex of LZRs and OZRs of 12-, 16-, and 20-weeks age processed for the immunohistochemistry against Neu-N protein, to identified cell body neurons. V, VI: fifth and sixth layers of the frontal cortex. Magnification 20X. Calibration bar: 50 μm. (**D**) Sections of the hippocampus (CA1 subfield) of LZRs and OZRs of different weeks of age processed for the immunohistochemistry against axonal NF. O: *stratum oriens*; P: pyramidal neurons; R: *stratum radiatum*. Calibration bar: 50 μm. Densitometric analysis of the frontal cortex (**E**) and hippocampus (**F**) in sections processed for axonal NF immunohistochemistry. Data are expressed in optical density unit (ODU). These are mean + S.E.M. * *p* < 0.01 vs. age-matched LZRs.

Immunoblots of the frontal cortex (Figure 3A) and hippocampus (Figure 3B) for Neu-N revealed a band at 46/48 kDa with a decreased expression of the nuclear protein in the older OZRs, suggesting a low neuronal vitality. Immunoblots of the frontal cortex (Figure 3A) and hippocampus (Figure 3B) for NF revealed a decreased expression of the protein in the brain of OZRs of different ages compared to LZRs. The number of Neu-N positive neurons in the layers of the frontal cortex showed a different pattern in OZRs compared to LZRs, with layers IV and V displaying the most relevant changes. The ANOVA and the post-hoc analysis indicate that the number of Neu-N positive neurons in these layers was significantly decreased in 20-weeks-old OZRs compared to age-matched LZRs (*p* = 0.01 both for the fifth and the sixth layer) (Table 2, Figure 3C). In the hippocampus, the CA1 and dentate gyrus represented two areas displaying the changes in Neu-N positive neurons in OZRs compared to the LZRs. In 20-week-old OZRs, the number of Neu-N positive cells was significantly lower (*p* = 0.0125) in the CA1 subfield and dentate gyrus (*p* = 0.011) compared to age-matched LZRs (Table 2).

In the frontal cortex, the NF immunoreactivity, which was localized mainly in the V and VI layers, showed a lower expression in OZRs of 12-, 16-, and 20 weeks of age compared to age-matched LZRs (*p* < 0.01) (Figure 3E). Sections processed for NF immunohistochemistry displayed a specific immunoreaction in the *stratum radiatum* of the CA1 subfield (Figure 3D), in the *hilus* and *molecular layer* of the dentate gyrus. Microdensitometric analysis of NF immunoreaction in the CA1 subfield revealed a low intensity in OZRs at different ages (*p* < 0.01) compared to LZRs (Figure 3F).

**Figure 4:**
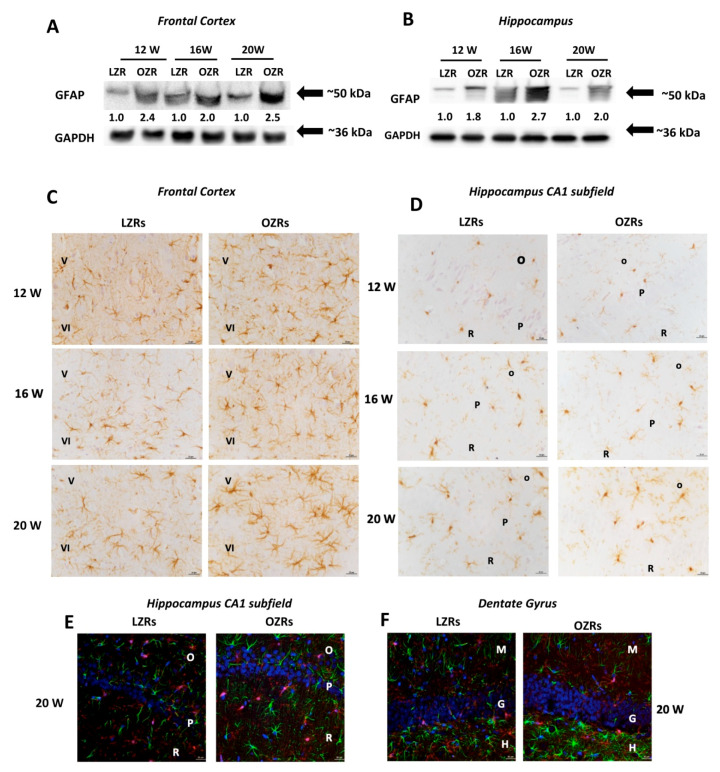
Glial Markers. Immunochemical analysis of the frontal cortex (**A**) and hippocampus (**B**) processed for anti-glial fibrillary acidic protein (GFAP). To investigate more than GFAP protein on the same blot, both membranes of the frontal cortex and hippocampus were stripped and incubated with anti-GAPDH antibody (loading control). Control images are re-used for illustrative purposes. The densitometric analysis of bands is expressed as the ratio between the optical density of protein and reference protein (GADPH) where the value for LZRs of different ages is set as 1. Blots are representative of one of three separate experiments. (**C**) Sections of the frontal cortex of LZRs and OZRs of different weeks of 12-, 16-, and 20-weeks age processed for the immunohistochemistry against the GFAP. V; IV: fifth and sixth layers of the frontal cortex. Magnification 40X. Calibration bar: 25 μm. (**D**) Sections of the hippocampus (CA1 subfield) of LZRs and OZRs of different weeks of age processed for the immunohistochemistry against ionized calcium-binding adaptor molecule 1 (Iba-1) to detect microglial cells O: *stratum oriens*; P: pyramidal neurons; R: *stratum radiatum.* Calibration bar: 25 μm. (**E**) Sections of the hippocampus (CA1 subfield) and (**F**) dentate gyrus of 20 weeks-old LZRs and OZRs processed for double immunohistochemistry against Iba-1 (Alexa Fluore 594) and GFAP (Alexa Fluore 488). DAPI was used to counterstain nuclei. O: *stratum oriens*; P: pyramidal neurons; R: *stratum radiatum*. Magnification 40X. Calibration bar: 25 μm.

GFAP immunoblots of the frontal cortex (Figure 4A) and the hippocampus (Figure 4B) revealed a specific band at 50 kDa with an increased expression of this marker of astrocytes in OZRs compared to LZRs. This increase was more pronounced in 20-week-old OZRs, indicating the development of a glial reaction in these animals (Figure 4A,B). Immunochemistry findings were confirmed by the immunohistochemical analysis, showing in the frontal cortex of OZRs a significant increase (*p* = 0.02) in the immunoreaction area of GFAP positive astrocytes (Table 3, Figure 4C). Hyper-reactive and hypertrophic astrocytes were observed in the V and VI layers near the *corpus callosum*, compared to LZRs of different ages. Similar results were obtained for the hippocampus, where increased number of astrocytes positive to GFAP was found compared to LZRs (Table 3). No significant increase in the size of perivascular astrocytes was found in OZRs, compared to the age-matched lean rats at the different weeks analyzed (*p* = 0.29; *p* = 0.28 and *p* = 0.75 for 12, 16 and 20 weeks respectively) (Table 3).

In the frontal cortex (V and VI layers) no reactive microglial cells were observed in older OZRs compared to lean littermates (Table 3). In the LZRs hippocampus, resting microglia was found in the white matter of the *stratum radiatum* of the CA1 subfield (Figure 4D), in the *hilus*, and *stratum molecular* of the dentate gyrus. In OZRs of 20 weeks of age, microglial cells with an obvious increase in the area of soma without arborization changes were found (Figure 4D). The post-hoc analysis revealed a significant increase in the area of the soma only in the different subfields of hippocampus of 20-weeks-old OZRs compared to the age-matched LZRs (*p* = 0.02) as shown in the Table 3. No phagocytic microglia were present in the white matter of the hippocampus in OZRs of different ages.

The sections processed for the double immunohistochemistry analysis against GFAP and ionized calcium-binding adaptor molecule 1 (Iba-1) analyzed at the confocal laser microscopy, showed in particular in the *stratum radiatum* of CA1 subfield (Figure 4E) and *hilus* of dentate subfields (Figure 4F) of 20-weeks old OZR a glial activation characterized by hypertrophic astrocytes and reactive microglia without colocalization of neuroglia arborizations (Figure 4E,F).

### 3.4. Analysis of Blood–Brain Barrier Markers Revealed an Impairment of BBB in Obese Rats

**Figure 5:**
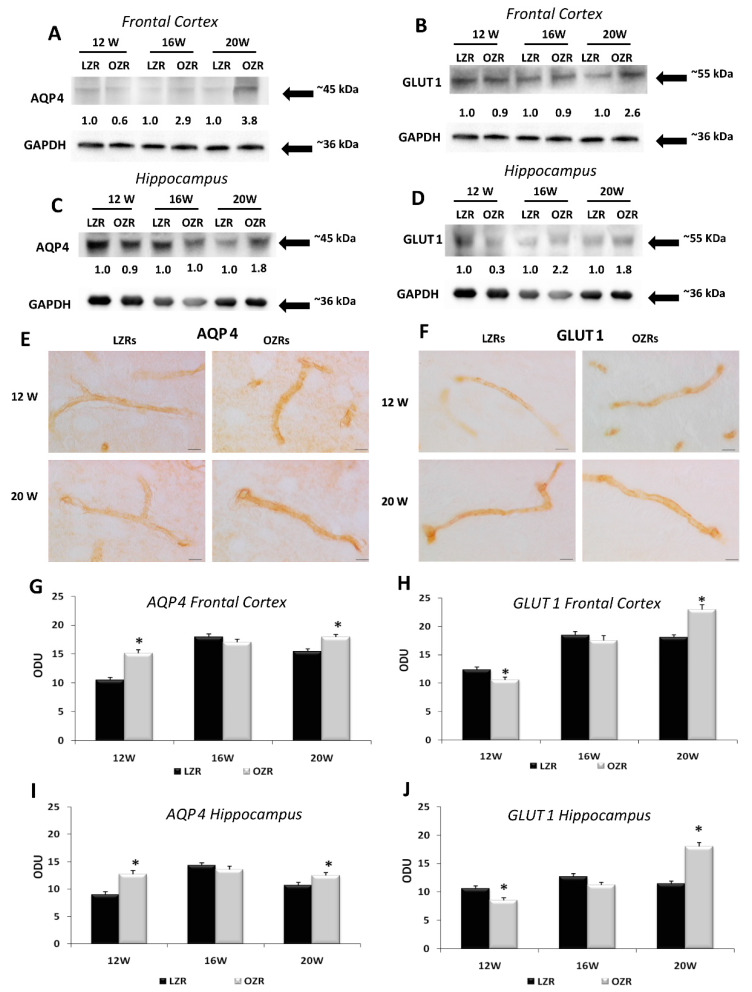
BBB Markers. Immunochemical analysis of the frontal cortex (**A**,**B**) and hippocampus (**C**,**D**) processed for aquaporin-4 (AQP4) (**A**,**C**) and glucose transporter-1 (GLUT1) (**B**,**D**). To investigate more than AQP4 and GLUT1 proteins on the same blot, both membranes of the frontal cortex and hippocampus were stripped and incubated with anti-GAPDH antibody (loading control). Control images are re-used for illustrative purposes. The densitometric analysis of bands is expressed as the ratio between the optical density of protein and reference protein (GADPH) where the value for LZRs of different ages is set as 1. Blots are representative of one of three separate experiments. Sections of the frontal cortex of LZRs and OZRs of 12 and 20 weeks of age processed for the immunohistochemistry against AQP4 (**E**) and GLUT1 (**F**). Magnification 63X. Calibration bar: 25 μm. Densitometric analysis of the frontal cortex (**G**,**H**) and the hippocampus (**I**,**J**) processed for AQP4 (**G**,**I**) and GLUT1 immunohistochemistry (**H**,**J**). Data are expressed in optical density unit (ODU). These are mean + SEM. * *p* < 0.05 vs. age-matched LZRs.

BBB markers AQP4 and GLUT1 were detected in the frontal cortex and hippocampus by Western blot analysis and immunohistochemistry. AQP4 and GLUT1 immunochemistry developed a band at 45 kDa (Figure 5A,C) and 55 kDa (Figure 5B,D) respectively, in the different brain areas examined. The AQP4 expression was higher in the hippocampus (Figure 5C) than in the frontal cortex (Figure 5A). A more intense immunoreaction was observed in OZRs compared to LZRs. No age-related changes of AQP4 immunoreactivity were observed in OZRs, whereas immunoreaction for the protein was decreased as a function of age in LZRs (Figure 5A,C). GLUT1 densitometric analysis showed an age-related up-regulation in OZRs both in the frontal cortex (Figure 5B) and hippocampus (Figure 5D).

Brain sections processed for AQP4 developed dark-brown staining around cerebral micro-vessels, confirming the localization of this water protein which represents a BBB marker in astrocytes foot processes (Figure 5E). Increased expression of AQP4 was found both in the frontal cortex (Figure 5G) and hippocampus (Figure 5I) of OZRs of different ages compared to age-matched LZRs, in particular in the 20 weeks-old OZRs, where the post-hoc analysis indicated a significant increase (*p* = 0.022) in the frontal cortex and in the hippocampus (*p* < 0.001). A similar localization was noticeable in sections processed for GLUT1 immunohistochemistry (Figure 5F). Compared to LZRs, OZRs have shown a decreased expression of GLUT1 in 12-weeks-old rats in frontal cortex (*p* = 0.012) and hippocampus (*p* = 0.021), while the immunoreactions density of GLUT1 was increased in 20-weeks-old in the frontal cortex (*p* < 0.001) than in the hippocampus (*p* < 0.001) (Figure 5H,J).

### 3.5. Behavioral Analysis: Anxiety-Like Behavior and Cognitive Impairment in Obese Rats

The overall ANOVA test revealed significant differences in the open field behavioral test between the two strains (*p* < 0.001). The post-hoc analysis confirmed that the OZRs showed a decrease of cumulative distance traveled, with an increment in the number of rearing (Table 4). OZRs rats also showed a decrease in the number of entries into the central zone compared with the age-matched LZRs rats (Table 4). In 20-week-old OZRs a significant decrease in the ratio between distance in entire open field and the central zone was observed (Table 4), indicating a condition of anxiety-like behavior. In the passive avoidance test, the statistical analysis revealed that only 20-weeks-old OZRs exhibited reduced retention latency time in the emotional learning task compared to the LZRs (*p* = 0.013). (Table 4).

## 4. Discussion

Obesity and MetS are identified as risk factors for adult-onset AD and vascular dementia [13,38]. Moreover, the combination of obesity, metabolic imbalances, and arterial hypertension occurring in MetS [39] have been associated with cognitive decline and their treatment is accompanied by a significantly decreased risk of dementia [40].

Because of these considerations, the availability of animal models of MetS can contribute to elucidate mechanisms of brain injury accompanying this disease and to identify possible treatments of it. Increasing evidence considers OZRs as a model to assess the MetS effects on organs structure and functions [28,29,41]. In terms of metabolic problems, OZRs are hyperlipidemic, hypercholesterolemic, and hyperinsulinemic and develop adipocyte hypertrophy and hyperplasia [21,22]. It was previously demonstrated that, on 17 day, the OZRs eat more compared with LZRs and hyperphagia increases during the growth period (approximately 16 weeks of life). It was revealed that the body composition of 14-weeks-old obese Zucker rats is 40% weight lipid. Furthermore, the systolic blood pressure in 8–12-weeks-old OZRs is lower than that in LZRs [21]. Based on these data, in our study, we sacrificed the rats at 12 weeks of age, when the OZRs established a condition of hyperphagia and body weight gain, but not yet the presence of overt hypertension. The difference in terms of blood pressure is not significant among the younger animals, but it was growing in obese condition, until reaching significance in 16-weeks-old OZRs [42].

The present study has shown lower expression of the nuclear and cytoskeletal components of nerve cells in the frontal cortex and hippocampus of 20 weeks-old OZRs. This presence validates the hypothesis of neurodegeneration in brain areas of rodents affected by obesity. NF protein is a particular constituent of the neuronal cytoskeleton. It is situated in the axons of neurons and represents a good marker of axonal damage [43]. The loss of NF immunoreactivity in the frontal cortex and the CA1 subfield of the hippocampus of OZRs is not accompanied by a parallel reduction of pyramidal neurons in the younger OZRs. Furthermore, the modifications of NF immunostaining demonstrate a breakdown of the cytoskeleton or damage rather than an axonal decline caused by nerve cell loss. Indeed, the reduction of NF immunoreactivity was more marked than nerve cell loss. The plausible explanation is that changes in neuronal environment occurring in a condition of hyperglycemia, hyperlipidemia, and oxidative stress induce cytoskeleton breakdown insufficient to cause neuronal death at least in the elapse of time observed in this study. Cytoskeleton breakdown appears in different neurodegenerative disorders affecting peripheral [44] and central [45] nervous system and could enhance neuronal vulnerability to hypoxia or ischemia [46], two conditions that may accompany hypertension related to metabolic dysfunction.

Another parameter indicative of damage in the brain of OZRs is the observation of astrogliosis. Astrocytes react to tissue damage by enhancing the GFAP synthesis, the intermediate filament protein specifically expressed in astrocytes [47]. For this reason, GFAP is an effective marker of astrogliosis, a phenomenon occurring in brains with neuronal loss, vascular and microvascular alterations. Moreover, in aging or in the presence of some diseases, the cerebral expression of GFAP and GFAP mRNA was higher [48,49]. Astrogliosis is common in the brain of spontaneously hypertensive rats and in streptozotocin-treated diabetic rats [49,50], two pathologies to some extent coexisting in OZRs and at the same time in a model of diet-induced obesity [30]. Data regarding the higher GFAP expression in OZRs are in agreement and corroborate the previous study [28]. The glial cells modify quickly their arrangement and function as a result of different events, for example, nerve cells impairment or loss, altered neuronal metabolic activity, and change of synaptic plasticity [47]. This phenomenon could be explained with the tendency of astrocytes to protect neuronal microenvironment in a condition of the suffering of nervous tissue caused by hyperglycemia and hypertension [47]. This hypothesis is supported by the findings of latent hyperglycemia [51], hypercholesterolemia [52], and oxidative stress [53] occurring in OZRs since the 12th week of age. Indeed, our results demonstrate a conditions of oxidative stress in brain area, related to the imbalance of antioxidant and pro-oxidant pathways, as demonstrated in the blood and the heart of OZRs [42].

Microglia represents the resident macrophage cells of the central nervous system and can engulf microorganisms, whole neurons [54], or parts of blood vessels [55] depending on their different activation state. Microglia also responds to activate signals by releasing intracellular mediators, such as cytokines and prostaglandins [56]. In a normal brain, microglia acts as a sensor of the equilibrium of the extracellular environment in response to changes or injury and transferring signals to the surrounding nerve cells or non-central nervous system immune cells. In response to harmful stimuli, microglia undergoes morphological and gene expression patterns change. Microglia in “resting state” reacts to an injury becoming a form with partial process de-ramification and process enlargement (activated state). This form can be further differentiated into different amoeboid forms with phagocyte activity characterized by the loss of cell processes [56]. Iba-1 immunoreactivity revealed in 20-week-old OZRs morphological changes of microglia with an increase of the area of soma, suggestive of an activated state, not yet evolving into amoeboid cells. At this age, obesity or related pathologies probably induced an inflammatory status, with microglial activation. As a consequence, chronic obesity in older animals might exacerbate this condition as demonstrated in OZRs of 30 weeks of age, which developed phagocytic microglia and related cytokines release, like interleukin 1-β and tumor necrosis factor-α [57].

Our results showed an increase of AQP4 in OZRs, similar to the results in the diet-induced obese rats [30], as well as in the animal model of hypertension [58]. Also, elevated expression of AQP4 was reported in the cerebral cortex of stroke-prone spontaneously hypertensive rat of 20 weeks and the spontaneously hypertensive rat of 32 weeks [58]. Despite the above results, the role of AQP4 in keeping BBB integrity has not been completely understood. AQP4 deletion alters BBB integrity and GFAP immunoreactivity in AQP4 null mice [59]. The increased expression of AQP4 was not correlated with the astrogliosis developed in the brain of OZRs rats. Our data indicated an increased expression of GFAP and an increasing size of astrocytes, a phenomenon not involving the perivascular astrocytes that selectively expressed AQP4.

In addition, our results demonstrated that in 12-weeks old obese rats, there is a decrease in the density of GLUT1 in brain micro-vessels. This could represent an adaptive phenomenon to avoid excessive glucose entry into neurons, following hyperglycemia. These data are in accordance with previous studies that have shown how in animal models of diabetes, induced by streptozotocin administration, there was a decrease in expression in the BBB of both GLUT1 and GLUT3 [60]. However, the modulation of glucose expression appears to be an extremely complex phenomenon. Indeed, the increased expression in 20-weeks old OZRs of the glucose transporter may be due to the inflammation [61]. Furthermore, in the brain of Zucker rats, microglial activation and increased Interleukin 1-β levels were demonstrated [57].

Performing basic behavioral tests, we observed an anxiety-like behavior and the impairment of learning and memory task in 20-weeks old OZRs. We found a significant decrease in the locomotor activity of OZRs since 12 weeks of observation, reflected by the lowest number of ambulatory and vertical counts compared to LZRs. Moreover, their reduced distance travelled in the entire open field and in the central zone plus the numbers of entries into the central area, revealed that OZRs showed limited exploration and anxiety-like behavior. Performing a passive avoidance task, to study learning and memory following a stressful stimulus (mild foot-shock), it was found a memory retention deficit only in older OZRs. In fact, the latency time to enter in the dark compartment, associated with foot-shock, was significantly less in OZRs than LZRs at 20 weeks.

Previously, disorders in insulin receptor signaling, neuronal glucose transporter trafficking, glucose uptake and consumption [62] were found in insulin deficient STZ-diabetic rats. In this latter [63] and in OZRs [64], cognitive deficits were reported; thus it was speculated that reduced insulin receptor activation is a shared feature contributing to cognitive impairment in these two models of diabetes [65]. Additional metabolic anomalies, such as the increase in plasma cortisol and triacylglycerides in obese animals were reported to be implicated in cognitive decline during aging [62,65] and supposed to contribute to the memory deficits [64].

Moreover, we observed in obese animals an anxious status started from the earliest age. Mouse model of MetS, namely the diabetic and obese db/db mice displayed as well increased anxiety-like behaviors in the open-field test [66]. Apart from the well-established role of the hippocampus in anxiety-like behaviors and spatial memory, behavioral alterations of db/db mice were associated with increased inflammatory cytokines (interleukin-1β, tumor necrosis factor-α and interleukin-6) and reduced expression of brain-derived neurotrophic factor (BDNF) in the hippocampus [66]. Instead, the possible role of leptin as a pathophysiologic mechanism in the generation of anxiety-like behavior is not completely understood [67].

These findings suggest that neurochemical and microanatomical changes, identified in this animal model, may have functional relevance. Further studies are needed to disclose the other mechanisms underlying such an association, using also other behavioral tests to clarify the correlation with cognitive alterations related to MetS. Since the hyperglycemia develops at 8 weeks of age in males Zucker rats but not in females [68,69,70], further study extended to females could clarify the role of different factors of MetS on brain impairment in both sexes.

## 5. Conclusions

The major finding of this work was the characterization of neuromorphological alterations due to brain injury related to the metabolic and functional changes affecting OZRs. Moreover, the presence of astrogliosis and microglia activation suggests the involvement of these cellular elements to protect neuronal microenvironment. These data may help to manage MetS progression by preventing the development of neurodegenerative processes that can evolve in dementia.

## Figures and Tables

**Table 1:** Primary and Secondary antibodies used in Western blot and immunohistochemistry (IHC).

Primary and Secondary Antibodies	Company Cat. No	Dilution Western Blot	Dilution IHC
**Anti Neuron-Specific nuclear protein (NEUronal Nuclei Neu-N)**	Monoclonal antibody Merk-Millipore Cat. MAB377	1:1000	1:500
**Anti-Neurofilament 200kDa, clone RT-97**	Monoclonal antibody Merk-Millipore Cat. MAB5262	1:1000	1:500
**Anti-Glial Fibrillary Acidic Protein (GFAP)**	Monoclonal antibody Merk-Millipore USA Cat. MAB3402	1:1000	1:500
**Anti-Glucose transporter 1 GLUT-1 (c-Terminus)** **clone 5B12.3**	Monoclonal antibody Merk-Millipore USA Cat. MABS132	1:500	1:500
**Ionized calcium binding adaptor molecule 1 (Iba-1)**	Thermofisher USA Cat. PA5-27436	----	1:200
**Anti Aquaporine-4 (AQP4)**	Monoclonal antibody Merk-Millipore USA AB3594	1:1000	1:500
**Anti-Mouse Biotinylated**	Polyclonal antibody Bethyl Lab. Cat. A90-116B	------	1:200
**Anti-Mouse HRP Conjugated**	Polyclonal antibody Bethyl Lab. Cat. A90-116P	1:5000	------
**Anti-Rabbit Biotinylated**	Polyclonal antibody Bethyl Lab. A120-101B	------	1:200
**Anti-Rabbit HRP Conjugated**	Polyclonal antibody Bethyl Lab. Cat. A120-101P	1:5000	------
**Anti-Mouse**	Alexa Fluor 488^®^	------	1:100
**Anti-Rabbit**	Alexa Fluor 594^®^	------	1:100

**Table 2:** Number of Neu-N positive neurons in frontal cortex and hippocampus of lean and obese Zucker rats at the different ages.

	12 Weeks		16 Weeks		20 Weeks	
	LZRs (*n* = 6)	OZRs (*n* = 6)	LZRs (*n* = 6)	OZRs (*n* = 6)	LZRs (*n* = 6)	OZRs (*n* = 6)
	***Frontal Cortex***					
**Fifth layer** **N/50,000 μm^2^**	21.6 ± 1.9	19.3 ± 1.7	19.5 ± 0.8	15.3 ± 2.2	20.0 ± 1.7	13.8 ± 0.7 *
**Sixth layer** **N/50,000 μm^2^**	40.3 ± 2.9	31.8 ± 2.8	39.7 ± 2.1	26.4 ± 3.9 *	42.8 ± 3.0	24.9 ± 2.0 *
	***Hippocampus***					
**CA1 subfield** **N/20,000 μm^2^**	65.0 ± 3.1	71.1 ± 3.2	71.3 ± 2.5	74.5 ± 4.8	76.3 ± 2.7	65.1 ± 3.2 *
**CA3 subfield** **N/20,000 μm^2^**	23.1 ± 1.2	24.1 ± 2.8	27.3 ± 2.1	29.4 ± 2.7	25.2 ± 1.2	24.6 ± 1.9
**Dentate gyrus** **N/20,000 μm^2^**	95.8 ± 2.3	96.1 ± 6.2	93.2 ± 4.2	95.7 ± 4.1	102.1 ± 2.6	87.5 ± 4.5 *

Data, expressed as number of positive neurons (N) for area, are the mean ± SEM. * *p* < 0.05 vs. age-matched LZRs.

**Table 3:** Mean immunoreaction area of GFAP and Iba-1 in the frontal cortex and hippocampus of lean and obese Zucker rats at the different ages.

	12 Weeks		16 Weeks		20 Weeks	
	LZRs (*n* = 6)	OZRs (*n* = 6)	LZRs (*n* = 6)	OZRs (*n* = 6)	LZRs (*n* = 6)	OZRs (*n* = 6)
	***Frontal Cortex***					
**Fifth layer** *Astrocytes immunoreaction area*	83.6 ± 5.5	114.8 ± 7.5 *	80.2 ± 5.6	100.1 ± 4.1 *	78.0 ± 4.2	113.3 ± 4.4 *
**Fifth layer** *Soma area of Iba-1 immunoreactive Cells*	40.3 ± 2.1	42.4 ± 1.8	39.7 ± 2.8	41.3 ± 1.2	42.5 ± 1.4	47.3 ± 1.7
	***Hippocampus***					
**CA1** *Astrocytes* *immunoreaction area*	83.9 ± 7.2	105.6 ± 4.5 *	78.7 ± 3.6	109.3 ± 4.8 *	89.6 ± 8.3	114.1 ± 6.1 *
**CA1** *Soma area of Iba-1 immunoreactive cells*	42.0 ± 3.9	41.6 ± 3.1	45.9 ± 4.9	55.1 ± 3.0	45.8 ± 0.8	60.1 ± 1.2 *
**CA3** *Astrocytes immunoreaction area*	83.1 ± 8.7	101.2 ± 5.2 *	93.5 ± 7.0	115.3 ± 6.7 *	101.1 ± 2.7	118.7 ± 2.7 *
**CA3** *Soma area of Iba-1 immunoreactive Cells*	40.7 ± 4.8	40.8 ± 3.1	42.5 ± 2.5	49.1 ± 5.6	46.0 ± 1.1	51.5 ± 2.2 *
**Dentate gyrus** *Mean immunoreaction area*	132.7 ± 10.8	130.2 ± 7.1	129.1 ± 8.0	130.4 ± 6.2	135.2 ± 5.5	130.4 ± 7.2
**Dentate gyrus** *Soma area of Iba-1 immunoreactive Cells*	40.6 ± 4.9	39.8 ± 2.2	42.2 ± 3.2	50.2 ± 4.9	44.5 ± 2.1	53.4 ± 3.2 *
	***Perivascular astrocytes***					
**Total Brain** *Mean immunoreaction area*	150.4 ± 9.3	164.1 ± 9.1	151.4 ± 5.6	140.7 ± 8.2	131.4 ± 6.4	128.7 ± 5.5

For astrocytes, data are expressed as mean immunoreaction area of GFAP positive astrocytes (μm^2^). For microglial cells data are the area of soma (μm^2^). Data are the mean ± SEM. * *p* < 0.05 vs. age-matched LZRs.

**Table 4:** Behavioural tests.

	Open Field					
	12 Weeks		16 Weeks		20 Weeks	
Parameters	LZRs (*n* = 6)	OZRs (*n* = 6)	LZRs (*n* = 6)	OZRs (*n* = 6)	LZRs (*n* = 6)	OZRs (*n* = 6)
**Tot.** **Dist. Trav. (cm)**	2741.6 ± 221.9	1307.4 ± 95.2 *	2160.1 ± 101.3	1100.1 ± 87.3 *	1910.9 ± 151.5	1009.2 ± 66.4 *
**Tot.** **Amb. Cnts**	1870.6 ± 156.2	764.3 ± 62.8 *	1956.0 ± 113.4	876.7 ± 90.5 *	1667.8± 142.5	789.5 ± 62.4 *
**Tot.** **Vert. Cnts**	94.4 ± 6.9	39.3 ± 3.7 *	78.3 ± 2.6	37.7 ± 5.2 *	83.5 ± 4.6	37.5 ± 4.5 *
**Central Zone. Dist. Trav.**	229.3 ± 7.6	103.3 ± 26.7 *	264.4 ± 22.6	91.9 ± 21.9 *	224.1 ± 36.1	56.1 ± 7.8 *
**Central zone Entries**	159.5 ± 9.7	60.2 ± 13.1 *	103.2 ± 9.8	34.5 ± 9.5 *	89.0 ± 12.1	21.5 ± 3.5 *
**% Dist. Trav.**	8.6 ± 0.6	8.4 ± 2.3	12.1 ± 0.5	9.2 ± 2.9	11.8 ± 2.0	5.7 ± 0.9 *
	**Passive Avoidance**					
**Latency time** **72 h after electric shock (s)**	176.3 ± 33.2	174.8 ± 30.7	171.3 ± 32.8	169.8 ± 30.8	177.3 ± 46.2	47.7 ± 16.2 *

Tot. Dist.Trav.: total distance travelled; Tot. Amb. Cnts: number of movements; Tot. Vert. Cnts: number of rearing; Central Zone Dist. Trav.: distance travelled on central zone; Central Zone Entries: number of entrances in the central zone; % Dist. trav: percentage ratio between the distance travelled in the central zone and the total area; Data are the mean ± SEM. * *p* < 0.05 vs. age-matched LZRs.

## Abbreviations

AD	Alzheimer’s disease
AQP4	aquaporin-4
BBB	blood brain barrier
GAPDH	glyceraldehyde 3-phosphate dehydrogenase
GFAP	glial fibrillary acid protein
GLUT1	glucose transporter protein-1
Iba-1	ionized calcium binding adaptor molecule 1
LZRs	lean Zucker rats
MetS	metabolic syndrome
NF	neurofilament-200 KDa
OZRs	obese Zucker rats
